# The effects of crossing ethnic boundaries on the autonomic nervous system in Muslim and Jewish young women in Israel

**DOI:** 10.1038/s41598-018-38290-z

**Published:** 2019-02-07

**Authors:** Diana Saadi, Keren Agay-Shay, Emanuel Tirosh, Izhak Schnell

**Affiliations:** 1 Porter School of Environmental Studies, Tel Aviv University, Department of Geography and Human Environment, Tel Aviv University, Tel Aviv, Israel; 2 The Azrieli Faculty of Medicine, Bar Ilan University, Zefat, Israel; 3 Bnei Zion Medical Center (emeritus), The Rappaport Family Faculty of Medicine, The Technion, Israel Institute of Technology, Haifa, Israel; 4 Department of Geography and Human Environment, Tel Aviv University, Tel Aviv, Israel

## Abstract

Exposure to alien inter-group environments can differently effect ethnic groups’ autonomous nervous system, measured by heart rate variability (HRV). Our aim was to evaluate the effect of crossing alien ethnic boundaries on heart rate variability in three selected types of environments. In a field experiment study, we test responses of 72 Muslim and Jewish women to exposure to alien ethnic environments. We measured their HRV in intra and inter-ethnic parks, town centers and residential neighborhoods in Arab and Jewish adjacent towns. The subjects stayed half an hour in each environment. Mixed models were used to evaluate the effects. The results show that for both groups more favorable HRV measurements were demonstrated in intra-ethnic environments as compared to their HRV once crossing ethnic boundaries. The strongest effect in frequency domain (LF/HF) in response to ethnic boundary crossing was observed in the park for Muslims (β:0.65, 95%CI: 0.60–0.70) and for Jews (β: 0.60, 95%CI: 0.57–0.63). Following the eruption of the uprising, the most significant increase in LF/HF in response to ethnic boundary crossing was demonstrated in parks (β: 0.66, 95%CI: 0.60–0.71). In conclusion, both groups are effected by boundary crossing but there are ethnic differences in the autonomic nervous system balance and in response to crossing alien ethnic boundaries. A further study is needed to understand the causes of these differences.

## Introduction

The identification of cities as environments that pose risks to health and of parks as restorative environments is well recorded in the literature. One study even concludes that a visit of 30 minutes a week in a park may reduce the risk to depression and high blood presser in 7 to 9 percent^1^. In many studies, the effect of the environment on the autonomous nervous system (ANS) is measured by heart rate variability (HRV)^2–10^. Recent literature review has suggested that the beneficial effects of green spaces might be modified by ethnicity^11^.

Only few studies, mainly in the USA, focus on ethnic differences in the response of ANS to environmental factors in different types of environments^12–24^. With the lack of evidence of genetic explanation for such differences^25^, two explanations are offered: one related to ethnic differences in lifestyles, and the other related to ethnic differences in coping with perceived inter-ethnic discrimination^17^. However, most of these studies fail to assess the contributable effect of these two mechanisms. These studies suggest that ethnic discrimination may be associated with reduced coping abilities with stress and increased risk to health as measured by HRV^26,27^.

Two (quasi) experimental studies support the hypothesis of an association between exposure to discrimination and HRV^28,29^. Wagner *et al*. studied the effects of self-reported discrimination on HRV and confirmed Hoggard *et al*. results that changes in high-frequency (HF) HRV were significantly associated with exposure to discrimination, while low-frequency (LF) HRV were not. They concluded that ethnic discrimination might be associated with parasympathetic reactivity.

Arabs in Israel, being a minority, may be considered as a discriminated ethnic group. In Israel, Arabs constitute about 22% of the population and about 85% of them are Muslims living in Arab-segregated towns. Several studies suggest that Arabs in Israel suffer from institutionalized and inter-personal discrimination^30–35^. Evidence from the experiences of Arab youngsters in Israel demonstrates that many are hesitant to cross ethnic boundaries, and they feel shyness in Jewish spaces in activities such as searching for work and leisure^36,37^. Therefore, we assume that crossing Arab–Jewish boundaries in Israel is associated with increased fear of exposure to ethnic discrimination.

Our main goal is to test the effects of crossing alien ethnic boundaries on HRV in three selected types of environments — hectic town centers, quiet residential environments, and town parks —between Muslim and Jewish young mothers in their fertile age. While all Jewish women were tested after the eruption of the uprising (knives terror attacks against Jews within Israel erupted in October by the Palestinians from the Occupied Territories), half of the Muslim women were tested before and half after the eruption of the uprising. Therefore, we focus on two comparisons: First, we compare the effects of crossing alien ethnic boundaries on HRV of Muslim and Jewish women after the eruption of the uprising. Second, we test differences in the changes of HRV before and after the eruption of the uprising among Muslim women in response to crossing ethnic boundaries. We hypothesize that: 1. both Jewish and Muslim women will experience less favorable measures of HRV once crossing inter-ethnic boundaries in all three environments. 2. In the case of Muslims, differences of HRV between intra-ethnic and inter-ethnic environments will increase in response to augmented inter-ethnic tensions.

## Results

**Figure 1:**
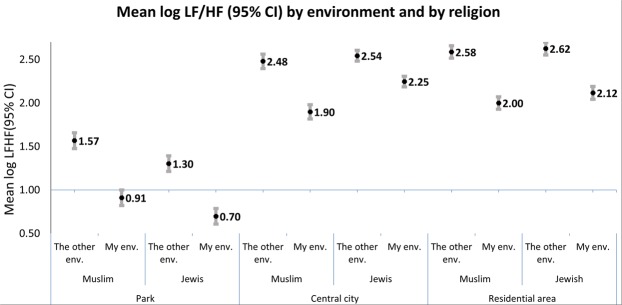
Estimated means of log LF/HF (95%Confidence Interval(CI)) by inter and intra-ethnic environments and women ethnicity. LF-low frequency; HF-high frequencies; LF/HF the ratio of LF-HF power, CI- Confidence Interval.

In analyzing frequency domain results, Fig. 1 and Table 1 depict the mean results of LF/HF by intra- and inter-ethnic environments, highlighting two main results. First, regardless of ethnicity, consistent lower LF/HF ratios in the two parks as compared to the LF/HF in town centers and residential environments were measured. Second, once the subjects crossed ethnic boundaries significant higher mean LF/HF in all three inter-ethnic environments were measured relative to their respective intra-ethic environments. Time domain analyses revealed similar trends. (Table 1).

**Table 1:** Estimated means of the HRV outcomes (95%Confidence Interval(CI)) and grand means for inter and intra-ethnic environments (park, town center and residential area), by ethnicity (Muslim and Jewish).

HRV outcomes		Parks		Town centers		Residential areas	
		Muslim	Jewish	Muslim	Jewish	Muslim	Jewish
Log LF/HF	Grand mean	1.24(1.15, 1.33)	1.00(0.91, 1.09)	2.19(2.11, 2.27)	2.39(2.33, 2.45)	2.29(2.22, 2.36)	2.37(2.30, 2.44)
	Inter-ethnic environment	1.57(1.48, 1.66)	1.30(1.21, 1.39)	2.48(2.40, 2.56)	2.54(2.48, 2.60)	2.58(2.51, 2.66)	2.62(2.55, 2.70)
	Intra-ethnic environment	0.91(0.82, 1.0)	0.70(0.61, 0.79)	1.90(1.81, 1.98)	2.25(2.18, 2.31)	2.00(1.93, 2.07)	2.12(2.04, 2.19)
Log HF	Grand mean	5.70(5.62, 5.77)	5.89(5.84, 5.93)	4.65(4.55, 4.74)	5.64(4.46, 6.82)	4.53(4.44, 4.61)	4.44(4.35, 4.53)
	Inter-ethnic environment	5.44(5.36, 5.52)	5.75(5.70, 5.80)	4.37(4.26, 4.47)	4.31(4.26, 4.37)	4.28(4.19, 4.37)	4.24(4.15, 4.33)
	Intra-ethnic environment	5.95(5.87, 6.03)	6.03(5.98, 6.08)	4.92(4.82, 5.03)	4.50(4.45, 4.56)	4.77(4.68, 4.86)	4.64(4.55, 4.73)
Log LF	Grand mean	6.93(6.87, 7.0)	6.89(6.84, 6.93)	6.83(6.79, 6.87)	6.80(6.78, 6.82)	6.82(6.78, 6.86)	6.81(6.78, 6.84)
	Inter-ethnic environment	7.01(6.94, 7.07)	7.05(7.00, 7.10)	6.85(6.80, 6.89)	6.86(6.83, 6.88)	6.87(6.83, 6.91)	6.86(6.83, 6.90)
	Intra-ethnic environment	6.86(6.79, 6.93)	6.73(6.68, 6.77)	6.82(6.78, 6.86)	6.75(6.73, 6.77)	6.77(6.73, 6.81)	6.76(6.72, 6.79)
Log SDNN	Grand mean	3.87(3.84, 3.91)	3.98(3.95, 4.00)	3.30(3.25, 3.35)	3.17(3.14, 3.20)	3.24(3.19, 3.29)	3.18(3.14, 3.22)
	Inter-ethnic environment	3.73(3.69, 3.77)	3.89(3.86, 3.91)	3.12(3.07, 3.18)	3.10(3.07, 3.14)	3.09(3.04, 3.14)	3.08(3.03, 3.12)
	Intra-ethnic environment	4.01(3.97, 4.05)	4.06(4.04, 4.09)	3.48(3.43, 3.53)	3.23(3.20, 3.26)	3.39(3.34, 3.44)	3.29(3.24, 3.33)
Log r-MSSD	Grand mean	3.60(3.57, 3.63)	3.68(3.65, 3.71)	3.03(2.96, 3.09)	2.85(2.79, 2.91)	2.93(2.87, 2.99)	2.88(2.81, 2.94)
	Inter-ethnic environment	3.51(3.48, 3.54)	3.59(3.56, 3.63)	2.74(2.67, 2.81)	2.69(2.63, 2.75)	2.64(2.58, 2.71)	2.64(2.57, 2.71)
	Intra-ethnic environment	3.69(3.66, 3.75)	3.77(3.73, 3.80)	3.31(3.24, 3.38)	3.02(2.96, 3.07)	3.21(3.15, 3.28)	3.11(3.05, 3.81)
Log HF_nu	Grand mean	3.09 (3.03, 3.16)	3.28 (3.21, 3.34)	2.3 (2.23, 2.37)	2.12 (2.06, 2.17)	2.21 (2.15, 2.27)	2.14 (2.07, 2.21)
	Inter-ethnic environment	2.84 (2.78, 2.91)	3.06 (2.99, 3.12)	2.03 (1.96, 2.11)	1.98 (1.92, 2.03)	1.95 (1.88, 2.01)	1.91 (1.84, 1.98)
	Intra-ethnic environment	3.34 (3.28, 3.41)	3.5 (3.43, 3.56)	2.56 (2.49, 2.64)	2.26 (2.2, 2.32)	2.47 (2.41, 2.54)	2.37 (2.31, 2.44)
Log LF_nu	Grand mean	4.33 (4.31, 4.36)	4.28 (4.25, 4.30)	4.49 (4.48, 4.50)	4.52 (4.51, 4.52)	4.5 (4.49, 4.51)	4.51 (4.50, 4.52)
	Inter-ethnic environment	4.41 (4.39, 4.44)	4.36 (4.33, 4.39)	4.52 (4.51, 4.53)	4.53 (4.52, 4.53)	4.53 (4.52, 4.54)	4.53 (4.53, 4.54)
	Intra-ethnic environment	4.25 (4.23, 4.28)	4.19 (4.17, 4.22)	4.46 (4.45, 4.47)	4.5 (4.50, 4.51)	4.47 (4.46, 4.48)	4.49 (4.48, 4.50)

LF-low frequency; HF-high frequencies; LF/HF the ratio of LF-HF power; LF_nu-Normalized power in LF band, HF_nu-Normalized power in HF band, a derived index that is computed by dividing HF by some suitable denominator representing the total relevant power; SDNN -the standard deviation of normal RR intervals; r-MSDD- the square root of the mean of the squared differences between adjacent normal RR intervals; HRV- heart rate variability. All outcomes were log transformed.

Some differences between the two ethnicities emerged mainly in response to exposure to park and town center environments. While Muslim women experienced higher LF/HF in parks relative to Jewish women (mean log LF/HF:1.24; 95%CI: 1.15–1.33 and 1.00; 95%CI: 0.91–1.09, respectively), Muslim women experienced lower LF/HF in town centers (mean log LF/HF:2.19; 95%CI: 2.11–2.27 and 2.39; 95%CI: 2.33–2.45, respectively) and in residential neighborhoods (mean log LF/HF:2.29; 95%CI: 2.22–2.36 and 2.37; 95%CI: 2.30, 2.44, respectively) relative to Jews (Table 1).

Table 2 depicts the magnitude of the changes in mean HRV in inter-ethnic environments compared to intra-ethnic environments for both the frequency domain and the time domain outcomes, for Jewish and Muslim women and for different urban environments. It appears from the frequency domain analysis, measured by LF,HF and LF/HF ratio, that Muslim women were more affected by boundary crossing relative to Jewish women, in town center(LF/HF,LF and HF), in residential area(LF/HF, HF) and in parks(HF and LF). Despite significant changes observed in both levels of HF and LF for crossing ethnic boundary in the parks, there are no significant differences between Muslim and Jewish women in LF/HF ratio. Both differences in the levels of LF and HF contributed to the changes observed in the levels of LF/HF in inter-ethnic environments relative to the corresponding intra-ethnic environments. These results were consistent for all three environments (Table 2). From comparing the normalized results of HF and LF (HF_nu, LF_nu) we observe a stronger effect of ethnic boundary crossing on HF. There were significant differences between Muslim and Jewish women in response to boundary crossing in LF/HF in town centers and residential neighborhoods mainly due to changes in HF (Table 2).

**Table 2:** Regression coefficient (95%Confidence Interval (CI)) for the change in mean HRV outcomes, in intra- ethnic environments compared to inter-ethnic environments, by environments (park, town centers and residential area) and by ethnicity (Muslim and Jewish women).

HRV outcomes	Parks		Town centers		Residential areas	
	Muslim	Jewish	Muslim	Jewish	Muslim	Jewish
Log LF/HF	0.65 (0.60, 0.70)	0.60 (0.57, 0.63)	0.58 (0.53, 0.62)	0.29 (0.26, 0.32)	0.58 (0.54, 0.62)	0.50 (0.48, 0.53)
Log HF	−0.51 (−0.56, −0.45)	−0.28 (−0.30, −0.25)	−0.55 (−0.61, −0.49)	−0.19 (−0.22, −0.15)	−0.48 (−0.53, −0.44)	−0.40 (−0.44, −0.36)
Log LF	0.14 (0.11, 0.17)	0.32 (0.29, 0.35)	0.02 (−0.01, 0.05)	0.1 (0.08, 0.12)	0.09 (0.07, 0.12)	0.10 (0.08, 0.13)
Log HF_nu	−0.5 (−0.54, −0.46)	−0.44 (−0.46, −0.42)	−0.53 (−0.56, −0.50)	−0.28 (−0.3, −0.26)	−0.53 (−0.56, −0.49)	−0.46 (−0.48, −0.44)
Log LF_nu	0.16 (0.14, 0.17)	0.17 (0.16, 0.18)	0.06 (0.05, 0.07)	0.02 (0.02, 0.03)	0.06 (0.05, 0.06)	0.05 (0.04, 0.05)
Log SDNN	−0.28 (−0.3, −0.25)	−0.17 (−0.19, −0.16)	−0.35 (−0.38, −0.31)	−0.12 (−0.14, −0.1)	−0.30 (−0.33, −0.27)	−0.21 (−0.23, −0.18)
Log r-MSSD	−0.17 (−0.20, −0.15)	−0.17 (−0.19, −0.15)	−0.57 (−0.6, −0.53)	−0.32 (−0.35, −0.29)	−0.57 (−0.60, −0.50)	−0.47 (−0.50, −0.44)

LF-low frequency; HF-high frequencies; LF/HF the ratio of LF-HF power; LF_nu-Normalized power in LF band, HF_nu-Normalized power in HF band, a derived index that is computed by dividing HF by some suitable denominator representing the total relevant power; SDNN -the standard deviation of normal RR intervals; r-MSDD- the square root of the mean of the squared differences between adjacent normal RR intervals; HRV- heart rate variability. All outcomes were log transformed.

The changes in the time domain results for crossing ethnic boundaries in the three environments, measured by SDNN and r-MSSD, were consistent with the frequency domain results. Changes in SDNN were stronger among Muslim women compared to Jewish women. Differences between Muslim and Jewish women in changes in r-MSSD as a result of crossing ethnic boundaries were significant in residential neighborhoods and town center (Table 2).

**Figure 2:**
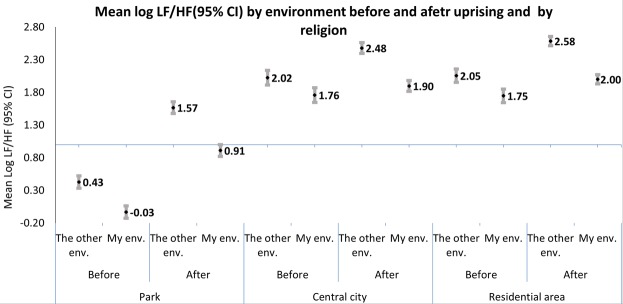
Estimated means of log LF/HF (95%Confidence Interval (CI)) by inter and intra-ethnic environment, before and after the eruption of the uprising. LF-low frequency; HF-high frequencies; LF/HF the ratio of LF-HF power, CI- Confidence Interval.

We measured changes in HRV in response to the uprising only for Muslim women. Figure 2 shows that mean results for LF/HF were consistently higher after the eruption of the uprising in all inter- and intra-ethnic environments. Table 3 shows that all these differences were significant with the exception of town centers. Following the uprising mean values of HF were lower in all three environments and in inter- and intra-ethnic environments respectively, LF values were higher only in parks and in inter-ethnic residential neighborhoods after the eruption of the uprising relative to their values before the uprising. These differences remained consistent for most cases also for the normalized outcomes (Table 3).

**Table 3:** Estimated means of the HRV outcomes (95%Confidence Interval (CI)), grand means and for inter and intra-ethnic environments (park, town center and residential area), for Muslim women, before and after the uprising.

HRV outcomes		Parks		Town centers		Residential areas	
		Before the uprising	After the uprising	Before the uprising	After the uprising	Before the uprising	After the uprising
Log LF/HF	Grand mean	0.20(0.11, 0.29)	1.24(1.15, 1.33)	1.89(1.78, 2.00)	2.19(2.11, 2.27)	1.90(1.80, 2.0)	2.29(2.22, 2.36)
	Inter-ethnic environment	0.43(0.33, 0.52)	1.57(1.48, 1.66)	2.02(1.91, 2.14)	2.48(2.4, 2.56)	2.05(1.95, 2.16)	2.58(2.51, 2.66)
	Intra-ethnic environment	−0.03(−0.13, 0.06)	0.91(0.82, 1.00)	1.76(1.65, 1.87)	1.9(1.81, 1.98)	1.75(1.64, 1.85)	2.00(1.93, 2.07)
Log HF	Grand mean	6.20(6.16, 6.24)	5.7(5.62, 5.77)	4.98(4.82, 5.15)	4.65(4.55, 4.74)	4.96(4.82, 5.09)	4.53(4.44, 4.61)
	Inter-ethnic environment	6.09(6.05, 6.14)	5.44(5.36, 5.52)	4.83(4.66, 5.00)	4.37(4.26, 4.47)	4.73(4.59, 4.87)	4.28(4.19, 4.37)
	Intra-ethnic environment	6.31(6.27, 6.36)	5.95(5.87, 6.03)	5.14(4.97, 5.31)	4.92(4.82, 5.03)	5.19(5.05, 5.32)	4.77(4.68, 4.86)
Log LF	Grand mean	6.4(6.35, 6.45)	6.93(6.87, 7.00)	6.88(6.81, 6.94)	6.83(6.79, 6.87)	6.86(6.81, 6.9)	6.82(6.78, 6.86)
	Inter-ethnic environment	6.52(6.46, 6.57)	7.01(6.94, 7.07)	6.85(6.79, 6.92)	6.85(6.8, 6.89)	6.78(6.74, 6.83)	6.87(6.83, 6.91)
	Intra-ethnic environment	6.28(6.22, 6.33)	6.86(6.79, 6.93)	6.9(6.83, 6.96)	6.82(6.78, 6.86)	6.93(6.88, 6.98)	6.77(6.73, 6.81)
Log SDNN	Grand mean	3.78(3.74, 3.83)	3.09(3.03, 3.16)	2.57(2.47, 2.66)	2.3(2.23, 2.37)	2.55(2.46, 2.64)	2.21(2.15, 2.27)
	Inter-ethnic environment	3.66(3.61, 3.71)	2.84(2.78, 2.91)	2.45(2.36, 2.55)	2.03(1.96, 2.11)	2.42(2.33, 2.51)	1.95(1.88, 2.01)
	Intra-ethnic environment	3.91(3.86, 3.96)	3.34(3.28, 3.41)	2.68(2.58, 2.78)	2.56(2.49, 2.64)	2.69(2.6, 2.78)	2.47(2.41, 2.54)
Log r-MSSD	Grand mean	3.98(3.94, 4.02)	4.33(4.31, 4.36)	4.46(4.44, 4.47)	4.49(4.48, 4.50)	4.46(4.44, 4.47)	4.5(4.49, 4.51)
	Inter-ethnic environment	4.09(4.04, 4.13)	4.41(4.38, 4.44)	4.48(4.46, 4.49)	4.52(4.51, 4.53)	4.48(4.47, 4.49)	4.53(4.52, 4.54)
	Intra-ethnic environment	3.87(3.83, 3.92)	4.25(4.23, 4.28)	4.44(4.42, 4.46)	4.46(4.45, 4.47)	4.44(4.42, 4.45)	4.47(4.46, 4.48)
Log HF_nu	Grand mean	3.78(3.74, 3.83)	3.09(3.03, 3.16)	2.57(2.47, 2.66)	2.3(2.23, 2.37)	2.55(2.46, 2.65)	2.21(2.15, 2.27)
	Inter-ethnic environment	3.66(3.61, 3.71)	2.84(2.78, 2.91)	2.45(2.36, 2.55)	2.03(1.96, 2.11)	2.42(2.33, 2.51)	1.95(1.88, 2.01)
	Intra-ethnic environment	3.91(3.86, 3.96)	3.34(3.28, 3.41)	2.68(2.58, 2.78)	2.56(2.49, 2.64)	2.69(2.6, 2.78)	2.47(2.41, 2.54)
Log LF_nu	Grand mean	3.98(3.94, 4.02)	4.33(4.31, 4.36)	4.46(4.44, 4.47)	4.49(4.48, 4.5)	4.46(4.45, 4.47)	4.5(4.49, 4.51)
	Inter-ethnic environment	4.09(4.04, 4.13)	4.41(4.38, 4.44)	4.48(4.46, 4.49)	4.52(4.51, 4.53)	4.48(4.47, 4.5)	4.53(4.52, 4.54)
	Intra-ethnic environment	3.87(3.83, 3.92)	4.25(4.23, 4.28)	4.44(4.42, 4.46)	4.46(4.45, 4.47)	4.44(4.42, 4.45)	4.47(4.46, 4.48)

LF-low frequency; HF-high frequencies; LF/HF the ratio of LF-HF power; LF_nu-Normalized power in LF band, HF_nu-Normalized power in HF band, a derived index that is computed by dividing HF by some suitable denominator representing the total relevant power; SDNN -the standard deviation of normal RR intervals; r-MSDD- the square root of the mean of the squared differences between adjacent normal RR intervals; HRV- heart rate variability. All outcomes were log transformed.

Table 4 depicts the magnitude of the changes in mean HRV for Muslim women, in inter-ethnic environments compared to intra-ethnic environments, before and after the eruption of the uprising, for both the frequency domain and the time domain outcomes, in the different urban environments. It shows that the magnitude of differences in LF/HF between intra- and inter-ethnic environments were highest in parks after the uprising and about similar in residential and central town environments. In all three environments, the magnitude of differences in LF/HF were higher after the eruption of the uprising than before. This was affected mainly by stronger negative effects in HF and HF nu while in LF and LF nu changes were inconsistent.

**Table 4:** Regression coefficient (95%Confidence Interval (CI)) for the change in mean HRV outcomes, in intra- ethnic environments compared to inter-ethnic environments, by environments (park, town centers and residential area), for Muslim women, before and after the uprising.

	Park		Town center		Residential street	
	Before the uprising	After the uprising	Before the uprising	After the uprising	Before the uprising	After the uprising
Log LF/HF	0.46 (0.39, 0.53)	0.66 (0.60, 0.71)	0.27 (0.23, 0.30)	0.58 (0.54, 0.62)	0.31 (0.27, 0.35)	0.59 (0.55, 0.62)
Log HF	−0.22 (−0.26, −0.19)	−0.51 (−0.56, −0.46)	−0.31 (−0.37, −0.25)	−0.56 (−0.62, −0.5)	−0.46 (−0.52, −0.4)	−0.49 (−0.54, −0.43)
Log LF	0.24 (0.20, 0.28)	0.15 (0.11, 0.18)	−0.04 (−0.08, −0.01)	0.02 (−0.01, 0.06)	−0.15 (−0.19, −0.11)	0.10 (0.07, 0.13)
Log HF_nu	−0.25(−0.28, −0.21)	−0.5(−0.54, −0.46)	−0.23(−0.26, −0.2)	−0.53(−0.56, −0.5)	−0.27(−0. 3, −0.25)	−0.53(−0.56, −0.49)
Log LF_nu	0.22(0.18, 0.25	0.16(0.14, 0.17)	0.04(0.03, 0.04)	0.06(0.05, 0.07)	0.04(0.04, 0.05)	0.06(0.05, 0.06)
Log SDNN	0.0 (−0.01, 0.0)	−0.28 (−0.31, −0.25)	−0.16 (−0.2, −0.13)	−0.35 (−0.39, −0.32)	−0.25 (−0.28, −0.22)	−0.3 (−0.33, −0.27)
Log r-MSSD	−0.07 (−0.1, −0.05)	−0.18 (−0.2, −0.16)	−0.19 (−0.23, −0.16)	−0.57 (−0.61, −0.53)	−0.23 (−0.26, −0.2)	−0.57 (−0.61, −0.53)

LF-low frequency; HF-high frequencies; LF/HF the ratio of LF-HF power; LF_nu-Normalized power in LF band, HF_nu-Normalized power in HF band, a derived index that is computed by dividing HF by some suitable denominator representing the total relevant power; SDNN -the standard deviation of normal RR intervals; r-MSDD- the square root of the mean of the squared differences between adjacent normal RR intervals; HRV- heart rate variability. All outcomes were log transformed.

For the time domain outcomes, both for SDNN and for r-MSSD, the effect of crossing ethnic boundary was stronger after the uprising compared to before the uprising. Significant differences between the effects for crossing ethnic boundaries were observed before and after the uprising in all the three environments for r-MSSD and in the park and the town center for SDNN.

## Discussion

Studies on ethnic differences in human response to exposure to urban environments show significant differences between ethnic groups. However, the underlying mechanism and the direction of the differences have not been substantiated. This study contributes to the debate by comparing HRV between intra-ethnic and inter-ethnic urban environments. The results confirm the hypothesis that exposure to ethnic alien environments leads to an effortful coping response and, consequently, to increased health risks as measured by HRV. The fact that the same subjects were exposed to comparable intra- and inter-ethnic environments enables the isolation of the effects of exposure to ethnic relation on HRV.

The fact that both Muslim and Jewish women experienced parallel deterioration in HRV once crossing ethnic boundaries leads to the conclusion that HRV responds to exposure to alien groups of others and not necessarily to direct exposure to discrimination. This conclusion is based on the assumption that wile Muslim women suffer from discrimination in Israeli society Jewish women do not suffer from parallel discrimination.

We argue that three results strengthen the empirical evidence for the relevancy of the exposure to alien ethnicities as affecting ANS function and consequently health status. First, is the fact that the results were consistent for the three types of environments: hectic central towns, quiet residential streets, and town parks. Second, is the fact that all five indices measured of both frequency and time domain analyses (LF, HF, LF/HF, SDNN, and r-MSSD) support each other in demonstrating the increased risk to health while crossing ethnic boundaries. Third, is the fact that increased inter-ethnic tensions, as the result of the Palestinian uprising, were associated with further increases in risk to health as measured by HRV, particularly in inter-ethnic environments.

Several studies have highlighted significant differences in HRV levels among ethnic groups. Most of these studies were performed among North-American ethnicities. They focused on comparison between Afro-American (AA) and European-American (EA) groups^12–16,18–24^.

Some of these studies argue that AAs experience higher levels of risk to health relative to EAs, measured by time domain indices of HRV. Wang *et al*.^25^ reported that after controlling for age, gender, and other covariates, young AA twins displayed higher levels of risk to health in comparison to similarly aged EA twin pairs, mainly due to lower levels of parasympathetic activity. No differences in heritability estimates for HRV were observed in this earlier investigation. A study by Liao *et al*.^37^ found lower levels of HF and higher levels of LF among AAs compared to EAs, adjusting for age and gender. A meta-analysis based on 17 empirical studies showed significant differences in levels of HRV between AAs and EAs, but these differences demonstrated inconsistent results^17^. The authors concluded that further studies are needed in order to understand associations between ethnicity and differences in HRV levels.

More controlled studies have supported the hypothesis of positive relationship between ethnic discrimination and risk to health measured by HRV. Hoggard *et al*.^28^ exposed a sample of AA and EA women to inter-group discriminating events in the first day of a two-day experiment. They found that exposure to inter-group discriminating events strongly decreased subjects’ levels of HF during the first day and more moderately during the second day. They concluded that exposure to discrimination affects cardiac activity in both the short and the long term. Hill *et al*. (2017) found that perceived discrimination accumulates along time to affect African Americans’ HRV even when they are not directly exposed to discrimination. Wagner *et al*.^29^ studied the effects of self-reported exposure to discrimination on women’s LF and HF levels. Our study confirms Wagner’s results by showing that both LF and HF levels were affected by exposure to ethnic alienation or discrimination. However, in line with Hoggard’s study, exposure to alienation or discrimination affected levels of HF more strongly than levels of LF. Comparing these results to our ones leads to the conclusion that HRV does not respond only to direct discrimination but to a more general factor of the quality of inter-ethnic relations. To large extent HRV differences between exposure to intra- and inter-ethnic environments may serve as quantitative indicators of inter-ethnic tensions. This result is further verified by the fact that HRV responds to changes in inter-ethnic tensions as demonstrated by the effect of the uprising on Muslim women.

Despite the indecisive conclusions of the meta-analysis by Hill *et al*.^28^, it seems that there is growing evidence of the effects of human exposure to ethnic alienation on increased health risks. Our results are in line with the studies that in one way or another attempted to isolate the contribution of alienation whether by discrimination or by other means from other possible effects related to ethnicity or environmental factors, at least among mothers in their fertile age. Our study adds a geographical dimension by showing that crossing ethnic boundaries between alien groups results in increased risk to health and that the increase in risk to health is affected by the degree of tensions between the groups.

Increase in risk to health in inter-ethnic environments is particularly strong in park environments, where subjects have high expectations to find a restorative environment. Schnell and Saadi^38^, who studied Arabs’ visits to parks, supported this result. It was found that with the lack of well-maintained parks in Arab towns, Arabs visiting parks in Jewish towns compensate for their sense of stress by joining the park in larger groups and by signifying ethnic markers to their immediate environment using loud voices and Arabic music. Participants reported that extrovert behavior helped them feel like they were in an Arab-controlled environment, thus, reducing their stress levels^38^.

The confirmation of the alienation hypothesis in this study does not exclude the contribution of alternative explanations to ethnic differences in coping with environmental risk factors. A study by Saadi *et al*. (forthcoming) showed that there were ethnic differences between Jewish and Muslim women in responses to environmental risks. While Muslim women responded to environmental risk factors by activating the parasympathetic system more intensively, Jewish women did so by activating the sympathetic autonomous system more intensively. Muslim women felt more stress in residential environments relative to town centers, while Jewish women experienced less favorable HRV in town centers than in residential environments. The discussion so far leads to the conclusion that differences in coping mechanisms with environmental risk factors in urban environments do exist between ethnicities, and they may be associated with lifestyle, cultural, and social (ethnic relations) factors. However, there is a need for more detailed studies that evaluate the relative contribution of each of these sources for ethnic differences in the functioning of the autonomous system. An example of a direct effect is differences in coping with crowded and noisy town centers as has been demonstrated by Muslim and Jewish women in this study. An example of an indirect effect is the tendency of Arabs to build up denser areas with less greenery relative to Jewish towns. The result is that Muslims are exposed to more stressing environments and consequently increased risk to health than Jews in the same area.

Our study may contribute to a wider debate about the effects of inter-ethnic encounters on ethnic relations. On the one hand, studies based on Allport’s (1955)^39^ hypothesis emphasize positive outcomes of inter-ethnic encounters, such as increased mutual understanding, friendships, positive attitudes toward others, reduction in stereotypes and prejudices, and increased creativity^40–44^. On the other hand, alternative studies emphasize that inter-ethnic encounters may increase racialization associated with loss of mutual trust, increased prejudices and stereotyping, anxiety, and stress depending on the socio-political structure of the community^45–49^. Our study supports the argument that the effects of inter-ethnic encounters in terms of HRV measurements are dependent on the socio-political context. Once subjects cross alien boundaries or are exposed to higher levels of political tensions, they are more vulnerable to health risks and stress. These more complex relations between socio-political structures and ethnic relations are also acknowledged by scholars like Valentine^48^ and Matejskova and Leitner (2011) based on qualitative research methods. However, we learn from this study that inter-ethnic relations may have direct effects on human health and that HRV measures may indicate the level of tension between the ethnic groups.

The current study employed an experimental setting that accounts for the possible bias that may occur in observational studies. However, our study had some limitations. The number of participants was small. This study focused on childbearing Muslim and Jewish women only. To generalize our findings, further evidence-based studies on a larger sample including various subject groups is required.

Further studies are needed in order to fully understand the effects of ethnicity on coping with environmental risk factors. We believe that such studies should focus on three goals: (1) A comparative analysis of HRV variables between various other ethnic groups employing intra- and inter-ethnic recordings similarly to the design employed in the present study to determine risk to health, and (2) employing other stress indices such as saliva steroids and questionnaires to establish the validity of the ANS response (3). To apply research strategies that will enable the isolation of the different causes for ethnic differences in coping with environmental risk factors.

## Conclusions

The present study lends support to the argument that people cope with different urban types of environments with different modulation of their ANS and that HRV indeed reflects such responses in a consistent manner. It also confirms that members of diverse ethnicities differ in their autonomic coping modes. While parks’ regardless of ethnicity are associated with a significant restorative power, Jewish and Muslim women appear to activate differently their autonomic systems while being exposed to urban centers and residential neighborhoods. Jewish women experience less favorable levels of HRV in all three environments, they experience higher risk to health in town center while Muslim women experience higher levels of risk to health in quiet residential neighborhoods. Therefore, it appears that both environmental and mental factors trigger an ethnically related differential modulation of the ANS as part of their coping mechanism.

## Research Methods

### Population and setting

We tested three groups of women (N = 72): 24 Jewish young women, 24 Muslim young women prior to the uprising, and 24 young Muslim women after the eruption of the uprising. All of them were non-smoking, non-drinking, and free of medications. We recruited all women from two small towns distanced 12 km from each other, and their vicinities of less than 80,000 inhabitants, each located in the Mediterranean climatic zone, one Arab — Nazareth —and one Jewish — Afula — both located at the lower Galilee. Nazareth is much more compact with less greenery and is topographically located about 200 m higher than Afula. Both towns have a town park and several smaller parks.

We conducted the within-subject field experiment, and to eliminate the effect of the order of sites, allocation of the visit order was randomized in 12 sessions of six women each. The sessions took place from January 2015 to February 2016. The first four sessions included only Muslim women, and we conducted them prior to the “knives uprising”. The other sessions took place between November 2015 and February 2016 and included four sessions of Muslim women and four sessions of Jewish women. All sessions started at 15:00 and ended not later than 20:00. Since our study focused on the immediate responses to environmental stimuli, the women stayed at each site for half an hour with a 15-minute break between sites. Between sites, the women stayed in an air-conditioned car at 22 °C. We chose sites in the hectic town center, quiet residential environment, and town park. This way, each woman visited six environments, three in her intra-ethnic town and three in her inter-ethnic town. The women wore the devices one hour before the experiment in order to adjust to them. One third of the subjects started the tests in each intra-ethnic site following the other two intra-ethnic sites and then the inter-ethnic sites in the same order.

### Outcomes measures

HRV was monitored by Polar 810i that automatically measured five minutes of R-R intervals. We calculated frequency domain index and time domain index^50^. The frequency domain analysis includes: HF frequently interpreted as a marker of the parasympathetic nervous system and is influenced by the respiratory rate. It is to a certain degree the same as the respiratory sinus arrhythmia and correlates with it. Parasympathetic regulation of the heart has a fast response after about 0.5 s and returns to baseline within1 s. LF is modulated both by the activity of the sympathetic and parasympathetic system. A high LF power often follows mental or physical stress. Sympathetic input leads to changes in heart rate, however, more slowly as after parasympathetic input, with a peak after about 4 s and return to baseline after about 20 s. The LF/HF ratio mirrors the general sympathetic/parasympathetic balance.

Time domain analysis measures the variation of the intervals between consecutive normal cardiac cycles. The SD of NN intervals (SDNN) is the most frequently used HRV parameter, formally the SD of all normal (“NN”) QRS distances. It correlates with total power (TP). The SD of the average NN intervals r-MSSD can be used both in short-term and long-term measurements. r-MSSD stands for the square root of the mean squared differences of successive NN interval. Importantly, time domain parameters depend on the length of the recording time. Longer periods generate more variability. The analysis of both types of indices, being derived with different calculation methodology, provides a more comprehensive short and longer term documentation of the ANS activity and in addition it facilitates reliability assessment of the results.

We calculated LF and HF using the frequency domain index in which LF ranged between 0.04 and 0.15 Hz, and HF ranged between 0.15 and 0.4 Hz. The LF range reflects the mixture of sympathetic and parasympathetic activation and the HF range gives a measure of vagal control. LF/HF is an index of the ratio of low-high frequency power that provides an assessment of the symphato-vagal balance. In addition, the normalized (or normalized unit) spectral indices defined as: LF_nu = LF/(LF + HF) and HF_nu = HF/(LF + HF) are calculated. It should be noted that although the validity of the relationship between sympathetic tone and LF power has been questioned (Reyes del Paso *et al*.^51^) an updated review suggests that LF indeed reflects mixed parasympathetic and sympathetic activity (Singh *et al*. 201 It should also be noted that although the validity of the relationship between sympathetic tone and LF power has been questioned^51^ an updated review suggests that LF indeed reflects mixed parasympathetic and sympathetic activity^52^.

The time domain was calculated from all normal RR intervals in the period of recording and was measured using the standard deviation of normal RR intervals (SDNN) and the square root of the squared differences between adjacent normal RR intervals (r-MSSD). SDNN reflects all the cyclic components responsible for variability in the period of recording and is considered an estimate of overall HRV, encompassing vagal and sympathetic influences; and the r-MSSD is considered as an estimate of short-term components of HRV, corresponding to parasympathetic activity. The Kubios HRV software version 2 (www.kubios.uku.fi) was used for the signals analysis including removal of artefacts.

### Statistical analyses

We produced 3,456 HRV measurements (72 subjects × 6 environments × 8 measurements per environment). Because of the multilevel nature of the data, thus accounting for repeated measures (i.e. HRV measurements for subject) we used mixed effects models. We entered subjects as random intercepts. We defined LF, HF, LF/HF, LF_nu, HF_nu, r-MSSD, and SDNN as the dependent variables. To estimate the contributing effect of the parasympathetic as compared to sympathetic activation on the changes in the autonomic balance in both ethnic groups the normalized frequencies (HF_nu and LF_nu) were employed. All outcomes were log transformed. Differences between intra- and inter-ethnic levels of the aforementioned HRV measures were defined as fixed effects (my environment vs. other environment). We created separate models for every type of environment (town center, residential, and park environments). We stratified the analyses by ethnicity in order to focus on the differences between Muslim and Jewish responses to the different types of environments and environmental exposures. In addition, for Muslim women we evaluated the β coefficients for the change in mean HRV outcomes in intra-ethnic environments compared to inter-ethnic environments, before and after the eruption of the uprising. We considered P values of 0.05 as statistically significant.

SPSS statistical software version 23 was used for all the analysis described above.

### Ethics

This study was approved by the Ethics Committee of Tel Aviv University and strictly followed their instructions. Before beginning the experiment, a full explanation of the research aim, the experimental procedure, and all measured indices was provided. Informed consent was obtained from all subjects.

## Data Availability

Data can be supplied upon request from corresponding author Izhak Schnell: schnell@post.tau.ac.il.

## References

[1] Shanahan DF (2016). Health Benefits from Nature Experiences Depend on Dose. Sci. Rep..

[2] Gladwell V, Kuoppa P, Tarvainen M, Rogerson M (2016). A Lunchtime Walk in Nature Enhances Restoration of Autonomic Control during Night-TimeSleep: Results from a Preliminary Study. Int. J. Environ. Res. Public Health.

[3] Lee J (2014). Influence of forest therapy on cardiovascular relaxation in young adults. Evid. Based. Complement. Alternat. Med..

[4] Lee J, Park B-J, Ohira T, Kagawa T, Miyazaki Y (2015). Acute Effects of Exposure to a Traditional Rural Environment on Urban Dwellers: A Crossover Field Study in Terraced Farmland. Int. J. Environ. Res. Public Health.

[5] Lee J (2011). Effect of forest bathing on physiological and psychological responses in young Japanese male subjects. Public Health.

[6] Ochiai H (2015). Physiological and Psychological Effects of Forest Therapy on Middle-Aged Males with High-NormalBlood Pressure. Int. J. Environ. Res. Public Health.

[7] Song C (2015). Effect of Forest Walking on Autonomic Nervous System Activity in Middle-Aged Hypertensive Individuals: A Pilot Study. Int. J. Environ. Res. Public Health.

[8] Song C (2013). Physiological and psychological effects of walking on young males in urban parks in winter. J. Physiol. Anthropol..

[9] Song C (2014). Physiological and psychological responses of young males during spring-time walks in urban parks. J. Physiol. Anthropol..

[10] Song C, Ikei H, Igarashi M, Takagaki M, Miyazaki Y (2015). Physiological and Psychological Effects of a Walk in Urban Parks in Fall. Int. J. Environ. Res. Public Health.

[11] Nieuwenhuijsen MJ, Khreis H, Triguero-Mas M, Gascon M, Dadvand P (2017). Fifty Shades of Green. Epidemiology.

[12] Choi J-B (2006). Age and Ethnicity Differences in Short-Term Heart-Rate Variability. Psychosom. Med..

[13] Hill LK, Siebenbrock A (2009). Are all measures created equal? Heart rate variability and respiration - biomed 2009. Biomed. Sci. Instrum..

[14] Sammito, S. & Böckelmann, I. Factors influencing heart rate variability. *Int*. *Cardiovasc*. *Forum J*. **6** (2016).

[15] Wang X (2009). Genetic influences on heart rate variability at rest and during stress. Psychophysiology.

[16] Zion AS (2003). Low arterial compliance in young African-American males. Am. J. Physiol. Circ. Physiol..

[17] Hill LK (2015). Ethnic differences in resting heart rate variability: a systematic review and meta-analysis. Psychosom. Med..

[18] Hinnant JB, Elmore-Staton L, El-Sheikh M (2011). Developmental trajectories of respiratory sinus arrhythmia and preejection period in middle childhood. Dev. Psychobiol..

[19] Jandackova VK, Scholes S, Britton A, Steptoe A (2016). Are Changes in Heart Rate Variability in Middle-Aged and Older People Normative or Caused by Pathological Conditions? Findings From a Large Population-Based Longitudinal Cohort Study. J. Am. Heart Assoc..

[20] Jones, V. Black Women Matter: Assessing Scales to Examine Minority Stress and Intersectional Microaggression. *SEWSA 2016 Intersect*. *New Millenn*. *An Assess*. *Cult*. *Power*, *Soc* (2016).

[21] Lampert R, Ickovics J, Horwitz R, Lee F (2005). Depressed autonomic nervous system function in African Americans and individuals of lower social class: a potential mechanism of race- and class-related disparities in health outcomes. Am. Heart J..

[22] Martin LA (2010). Ethnicity and Type D personality as predictors of heart rate variability. Int. J. Psychophysiol..

[23] Muntner P (2015). Racial Differences in Abnormal Ambulatory Blood Pressure Monitoring Measures: Results From the Coronary Artery Risk Development in Young Adults (CARDIA) Study. Am. J. Hypertens..

[24] Sloan RP (2008). Cardiac autonomic control and the effects of age, race, and sex: the CARDIA study. Auton. Neurosci..

[25] Wang X, Thayer JF, Treiber F, Snieder H (2005). Ethnic differences and heritability of heart rate variability in African- and European American youth. Am. J. Cardiol..

[26] Chao RC-L, Longo J, Wang C, Dasgupta D, Fear J (2014). Perceived Racism as Moderator Between Self-Esteem/Shyness and Psychological Distress Among African Americans. J. Couns. Dev..

[27] Min, Y.-I. *et al*. Abstract P084: Cardiovascular Disease Burden in African Americans: The Jackson Heart Study. *Circulation* **133**, (2016).

[28] Hoggard LS, Hill LK, Gray DL, Sellers RM (2015). Capturing the cardiac effects of racial discrimination: Do the effects “keep going”?. Int. J. Psychophysiol..

[29] Wagner J, Lampert R, Tennen H, Feinn R (2015). Exposure to Discrimination and Heart Rate Variability Reactivity to Acute Stress among Women with Diabetes. Stress Heal..

[30] Bar-Tal D, Labin D (2001). The effect of a major event on stereotyping: terrorist attacks in Israel and Israeli adolescents’ perceptions of Palestinians, Jordanians and Arabs. Eur. J. Soc. Psychol..

[31] Berger R, Benatov J, Abu-Raiya H, Tadmor CT (2016). Reducing prejudice and promoting positive intergroup attitudes among elementary-school children in the context of the Israeli–Palestinian conflict. J. Sch. Psychol..

[32] Goren C, Neter E (2016). Stereotypical thinking as a mediating factor in the association between exposure to terror and post-traumatic stress disorder symptoms among Israeli youth. Anxiety, Stress. Coping.

[33] Maoz I (2011). Does contact work in protracted asymmetrical conflict? Appraising 20 years of reconciliation-aimed encounters between Israeli Jews and Palestinians. J. Peace Res..

[34] Niwa EY (2016). Negative Stereotypes of Ethnic Outgroups: A Longitudinal Examination Among Palestinian, Israeli Jewish, and Israeli Arab Youth. J. Res. Adolesc..

[35] Walther JB, Hoter E, Ganayem A, Shonfeld M (2015). Computer-mediated communication and the reduction of prejudice: A controlled longitudinal field experiment among Jews and Arabs in Israel. Comput. Human Behav..

[36] Schnell, I. & Haj-Yahya, N. Arab integration in Jewish-Israeli social space: does Schnell, Izhak, and Nasreen Haj-Yahya. 2014. “Arab Integration in Jewish-Israeli Social Space: Does Commuting Make a Difference?” Urban Geography 35 (7). Routledge: 1084–1104 https://doi.org/10.1080/0. *Urban Geogr*. **35**, 1084–1104 (2014).

[37] Liao D (1995). Age, race, and sex differences in autonomic cardiac function measured by spectral analysis of heart rate variability—The ARIC study. Am. J. Cardiol..

[38] Schnell, I. & Saadi, D. The social role of the Arab city park. In Sustainability in the Design of Gardens in Israeli Cities. in *Sustainability in the Design of Gardens in Israeli Cities* (eds Schnell, I., Rosenberg, A. & Ronen, G.) 249–284 (Pardes [in Hebrew], 2014).

[39] Allport, G. W. (Gordon W. *Becoming: basic considerations for a psychology of personality*. (Yale University Press,1955).

[40] Dixon JC, Rosenbaum MS (2004). Nice to Know You? TestingContact, Cultural, and Group Threat Theories of Anti-Black and Anti-Hispanic Stereotypes*. Soc. Sci. Q..

[41] Emerson MO, Kimbro RT, Yancey G (2002). Contact Theory Extended: The Effects of Prior Racial Contact on Current Social Ties. Soc. Sci. Q..

[42] Jehn KA, Northcraft GB, Neale MA (1999). Why Differences Make a Difference: A Field Study of Diversity, Conflict, and Performance in Workgroups. Adm. Sci. Q..

[43] Pettigrew TF, Tropp LR (2006). A meta-analytic test of intergroup contact theory. J. Pers. Soc. Psychol..

[44] Pettigrew TF, Tropp LR (2008). How does intergroup contact reduce prejudice? Meta-analytic tests of three mediators. Eur. J. Soc. Psychol..

[45] Matejskova T, Leitner H (2011). Urban encounters with difference: the contact hypothesis and immigrant integration projects in eastern Berlin. Soc. Cult. Geogr..

[46] Stephan WG, Stephan CW (1985). Intergroup Anxiety. J. Soc. Issues.

[47] Stephan WG, Ybarra O, Bachman G (1999). Prejudice Toward Immigrants1. J. Appl. Soc. Psychol..

[48] Valentine G (2008). Living with difference: reflections on geographies of encounter. Prog. Hum. Geogr..

[49] Van Zomeren M, Fischer AH, Spears R (2007). Testing the Limits of Tolerance: How Intergroup Anxiety Amplifies Negative and Offensive Responses to Out-Group-InitiatedContact. Personal. Soc. Psychol. Bull..

[50] Sztajzel, J. Heart rate variability: a noninvasive electrocardiographic method to measure the autonomic nervous system (2004). 10.4414/smw.2004.10321 15517504

[51] Reyes del Paso GA, Langewitz W, Mulder LJM, van Roon A, Duschek S (2013). The utility of low frequency heart rate variability as an index of sympathetic cardiac tone: A review with emphasis on a reanalysis of previous studies. Psychophysiology.

[52] Singh N (2018). Heart Rate Variability: An Old Metric with New Meaning in the Era of using mHealth Technologies for Health and Exercise Training Guidance. Part One: Physiology and Methods. Arrhythmia Electrophysiol. Rev..

